# Dual-Route Model of the Effect of Head Orientation on Perceived Gaze Direction

**DOI:** 10.1037/a0036151

**Published:** 2014-04-14

**Authors:** Yumiko Otsuka, Isabelle Mareschal, Andrew J. Calder, Colin W. G. Clifford

**Affiliations:** 1 School of Psychology, The University of Sydney, Sydney, Australia; 2 The University of Sydney, Sydney, Australia and School of Chemical and Biological Sciences, Department of Psychology, Queen Mary University of London, London, United Kingdom; 3 MRC Cognition and Brain Sciences Unit, Cambridge, United Kingdom; 4 School of Psychology, The University of Sydney, and Australian Centre of Excellence in Vision Science, The University of Sydney, Sydney, Australia

**Keywords:** gaze perception, cue combination, head orientation, Wollaston effect, dual-route model

## Abstract

Previous studies on gaze perception have identified 2 opposing effects of head orientation on perceived gaze direction—1 repulsive and the other attractive. However, the relationship between these 2 effects has remained unclear. By using a gaze categorization task, the current study examined the effect of head orientation on the perceived direction of gaze in a whole-head condition and an eye-region condition. We found that the perceived direction of gaze was generally biased in the opposite direction to head orientation (a repulsive effect). Importantly, the magnitude of the repulsive effect was more pronounced in the eye-region condition than in the whole-head condition. Based on these findings, we developed a dual-route model, which proposes that the 2 opposing effects of head orientation occur through 2 distinct routes. In the framework of this dual-route model, we explain and reconcile the findings from previous studies, and provide a functional account of attractive and repulsive effects and their interaction.

Accurate perception of another person’s gaze direction plays an important role in human communication. From an examination of the external morphology of the eyes in nearly half of all extant primate species, Kobayashi and Kohshima (1997, 2001) reported that human eyes have a unique morphology among primates, in that they have a widely exposed white sclera contrasting against a dark-colored iris and pupil. They proposed that the white sclera of the human eye is an adaptation to facilitate the signaling of gaze direction to others, whereas the dark pigmented sclera around the iris in nonhuman primates is an adaptation to camouflage gaze direction from other individuals and predators. Indeed, earlier psychophysical investigations have revealed the highly accurate nature of human gaze perception (Cline, 1967; Gibson & Pick, 1963).

However, previous studies have also reported that the perceived direction of gaze is influenced by various properties of the face. For example, gaze direction is shown to be biased to be perceived as “direct” when the eyes are not clearly visible (Mareschal, Calder, & Clifford 2013a; Martin & Jones, 1982; Martin & Rovira, 1981) or when the face is showing a smiling or angry expression (e.g., Ewbank, Jennings, & Calder, 2009; Lobmaier, Tiddeman, & Perrett, 2008; Martin & Rovira, 1982; Slepian, Weisbuch, Adams, & Ambady, 2011). Further, numerous studies have reported an influence of head orientation on gaze perception (e.g., Anstis, Mayhew, & Morley, 1969; Gamer & Hecht, 2007; Gibson & Pick, 1963; Langton, 2000; Langton, Honeyman, & Tessler, 2004; Ricciardelli & Driver, 2008; Seyama & Nagayama, 2005; Todorović, 2006, 2009; Wollaston, 1824). Studies measuring reaction times (RTs) for judgments of gaze direction are generally consistent in showing that RT in a speeded task is facilitated when eye gaze and head orientation are in the same direction compared with when they are in inconsistent directions (Langton, 2000; Ricciardelli & Driver, 2008; Seyama & Nagayama, 2005).

Although many studies have examined the influence of the head orientation on perceived gaze direction, they have been inconsistent about the direction of bias induced by the head orientation. In a pioneering study, Gibson and Pick (1963) used real human faces as the stimuli for their exploratory experiment, and reported that perceived gaze direction was consistently biased opposite to the head orientation (repulsive effect). A similar effect of head orientation has been reported not only using real human faces, as in Gibson and Pick, but also using an artificial eye model (Anstis et al., 1969) and realistic 3D graphic faces (Gamer & Hecht, 2007). Finding a similar repulsive effect of head orientation for both real faces and for artificial eyes, Anstis et al. (1969) noted that turning the head with gaze fixed on a given point (e.g., directly ahead) changes the relative amount of visible white (sclera) on either side of the iris. As the head rotates to the right, for example, the relative amount of visible sclera on the right side of the iris increases, just like when eye direction shifts toward the left. Anstis et al. (1969) argued that such effects support the notion that “judgments of direction of gaze are determined principally by the position of the pupil in the visible part of the eye” (p. 489). By using facial images in which one of the eyes was occluded as well as fully visible facial images, Noll (1976) reported that the repulsive effect occurred when both eyes or the nearer eye of a turned head was visible, while the perception of the gaze direction from the further eye was close to veridical. More recently, Gamer and Hecht (2007) reported that the point of subjectively direct gaze was generally biased toward the head rotation, especially at closer viewing distance, which is again consistent with the repulsive effect (because a slightly leftward gaze deviation [e.g., 5°] will appear direct if it is being repulsed away from its veridical deviation toward direct [0°] when the head is oriented leftward).

Unlike Gibson and Pick (1963) and others (Anstis et al., 1969; Gamer & Hecht, 2007; Noll, 1976), Cline (1967) reported that gaze direction was constantly biased toward the head orientation (attractive effect) when the head was rotated rightward by 30°. Such an effect is easily observable in the demonstration by Wollaston (1824). From the drawing of a face oriented leftward with direct gaze (Figure 1, left), Wollaston produced another face by inserting the same eyes into a drawing of the same individual with his head oriented to the right (Figure 1, right). Wollaston noted that although the first figure appears to have direct gaze, the latter seems to be looking to the right of the viewer. A similar demonstration was provided by Gibson and Pick (1963, p. 338–339), in which the perceived gaze direction of schematic eyes varies depending whether the eyes are shown alone or in the context of an angled face. Gibson and Pick noted that, in the latter case, the perceived gaze direction is attracted toward the orientation of the face. Based on that demonstration, they proposed that, except for the special case of frontal head orientation, information given within the eyes is insufficient to determine the direction of gaze. These observations of an attractive effect have been supported by several psychophysical studies (e.g., Langton et al., 2004; Maruyama & Endo, 1983; Todorović, 2006, 2009). Thus, counter to the notion of Anstis et al. (1969) that the influence of head orientation on perceived gaze direction is determined by its effect on the visible part of the eye, the findings from these studies suggest that head orientation has a direct attractive influence on perceived direction of gaze.[Fig-anchor fig1]

From photographs of faces, Langton et al. (2004) created stimuli similar to Wollaston (1824) that had identical eyes (either direct of averted) placed in the context of either congruent or incongruent head orientation (frontal or angled). Langton et al. found that gaze judgment was more accurate for the condition with congruent gaze and head direction than the incongruent condition, demonstrating that head orientation modulates perceived gaze direction with the identical eyes. They further reported that the modulation of head outline or nose angle alone could induce a similar effect. Using schematic facial image, Maruyama and Endo (1983) and Todorović (2009) showed that simple lateral displacement of the internal facial features relative to the head outline could also induce an attractive effect of head orientation. Todorović (2006) manipulated the relative position of the iris within the eyes, as expressed by iris eccentricity (distance of the pupil center from the center of the eye opening), and head rotation independently in realistic synthetic facial images. He asked his subjects to judge whether a face was directly gazing at them or not across various iris eccentricities and head rotations. Todorović (2006) found that the peak of the “direct” response distribution across the iris eccentricity shifted opposite to the direction of head rotation, suggesting the attractive influence of head orientation. By using schematic facial images, Todorović (2009) independently manipulated iris eccentricity as a cue for eye deviation and face eccentricity (position of internal facial features relative to the outline head contour) as a cue for head rotation. Across various tasks, including categorical judgment of gaze direction as left/right or “direct”/averted, and the judgment of the angle of the looker’s line of regard, Todorović (2009) found that the perceived gaze direction was consistently attracted toward the direction in which face eccentricity shifted. Finally, using photographs of faces in two orientations (frontal and oriented leftward), Kluttz, Mayes, West, and Kerby (2009) measured the perceived direction of gaze for images containing eyes in isolation or placed in a whole face context in either the original or different orientation, as well as the orientation of head with closed eyes. Kluttz et al. reported that both gaze direction and head orientation were underestimated in isolation, and that the judgment of the gaze direction improved when the eyes were shown in the whole face context. Their results also show a general tendency for head orientation to have an attractive influence when the results for identical eyes placed in differential facial orientation contexts are considered. Further, their data show that the improvement of gaze direction estimation in the whole face generally occurs as an attractive shift of the perceived gaze direction toward the head orientation.

When considered together, two opposing effects of head orientation on perceived gaze direction have been identified—one repulsive and the other attractive. However, the relationship between these two effects is not clearly understood. This is perhaps because it was difficult to apply an integrative framework for these effects, given that each study identified only one of the two effects. It is notable that the studies reporting the repulsive effect used stimuli such as real faces or facial images, based on a 3D model that included a change in the visible part of the face and eyes along with the change in head orientation. On the other hand, most studies reporting the attractive effect placed identical eyes in varying head orientation contexts, thereby precluding any change in the visible part of the eyes across the change in head orientation. These differences in stimulus manipulation may account for the inconsistent findings. Our study goes beyond those conducted previously by measuring the effect of head orientation in an eye-region condition, in which little or no information about head orientation is available, as well as in a whole-head condition, in which the head is fully visible. Here, we propose that the two opposing effects of head orientation on perceived gaze direction occur through two distinct routes. The repulsive effect would primarily depend on the change in the information from the eye region along with head rotation, as proposed by Anstis et al. (1969). The attractive effect represents a direct influence of head orientation on gaze perception, as first reported by Wollaston (1824).

Perception of gaze direction in most situations would involve the effect of head orientation from both of these two routes. As discussed by Anstis et al. (1969), information in the eye region inevitably changes according to head rotation, which gives rise to the repulsive effect. When visible, however, the direct influence of head orientation (i.e., attractive effect; Langton et al., 2004; Maruyama & Endo, 1983; Todorović, 2006, 2009) would compensate for the repulsive effect induced from the angled eye region and reduce the error in the resultant perceived gaze direction. This suggests that the seemingly illusory shift of gaze direction in the demonstration by Wollaston (1824) reflects a functional property of our gaze processing that helps to maintain the perceived direction of gaze closer to veridical in spite of changes in head orientation. In the current study, we explicitly tested this possibility by examining perceived gaze direction across various head orientations in a whole-head condition and in an eye-region condition using a gaze categorization task (Ewbank et al., 2009; Mareschal, Calder, Dadds, & Clifford, 2013b; Stoyanova, Ewbank, & Calder, 2010). We hypothesized that a greater repulsive effect of head orientation would occur for the eye-region condition.

## Experiment

### Method

#### Participants

Twenty naïve observers (10 male and 10 female) served as subjects (mean age = 18.95 years; *SD* = 1.99 years). All had normal or corrected normal vision. All experiments adhered to the Declaration of Helsinki (2008) guidelines and were approved by the University of Sydney Human Research Ethics Committee.

#### Apparatus

A Dell OptiPlex 990 computer running Matlab (MathWorks, Inc.) was used for stimulus generation, experiment control, and recording subjects’ responses. The programs controlling the experiment incorporated elements of the PsychToolbox (Brainard, 1997). Stimuli were displayed on a Viewsonic Graphics Series G90f (1024 × 768 pixels) driven by the computer’s built-in NVIDIA GeForce GTS 240 graphics card. The display was calibrated using a photometer and linearized using look-up tables in the software. At the viewing distance of 57 cm, 1 pixel subtended 2 arcmin.

#### Stimuli

**Figure 2 fig2:**
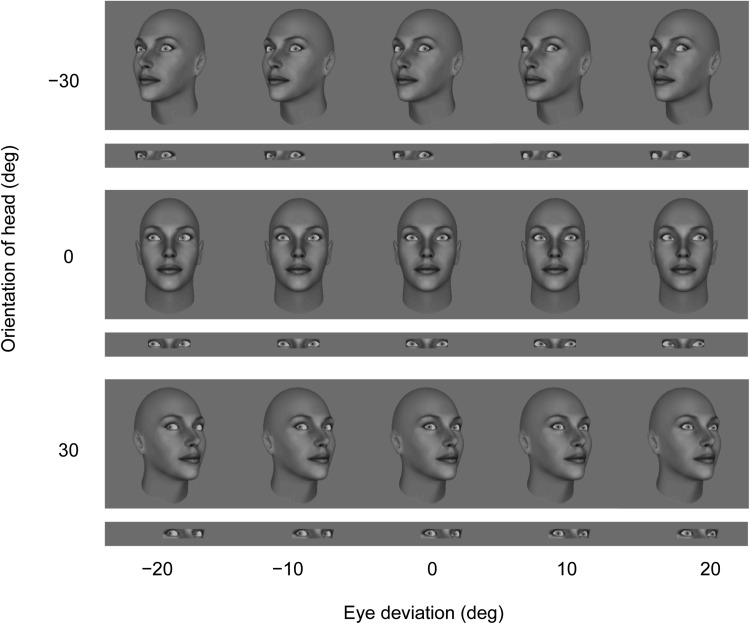
Example stimuli from the whole-head display condition and the corresponding stimuli in the eye-region display condition (shown in thin stripes).

Four gray-scale synthetic neutral faces (FaceGen Modeler 3.5), two male faces and two female faces, were used as the stimuli. The faces subtended 19° × 11° and were viewed at 57 cm in a dimly lit room. As in previous studies from this laboratory (e.g., Mareschal et al., 2013a, 2013b), the original eyes in the faces were replaced using Gimp software by gray-scale eye stimuli created using Matlab in order to control the deviation of the eyes. The deviation of each eye was independently controlled using Matlab procedures, giving us precision down to the nearest pixel for horizontal eye rotations. In the eye-region display condition, facial images were masked except for a rectangular 6.5° × 1.5° region around both eyes. All images were shown against a medium gray background. Examples of the stimuli in the whole-head condition and those in the eye-region condition are shown in Figure 2.[Fig-anchor fig2]

#### Procedure

The observers’ task was to indicate whether the direction of gaze was averted to the left, was direct, or was averted to the right using key-presses “j,” “k,” and “l,” respectively. Participants were given both written and verbal instruction as follows,

On each trial, you will be shown either an image of a face, or of eyes only. Your task is to judge the gaze direction, whether it is looking to YOUR LEFT, looking STRAIGHT AT YOU, or looking to YOUR RIGHT.

Each stimulus was presented for 500 ms, followed by a gray screen that lasted 300 ms, during which no response was recorded. The next trial was only initiated after a response was made following the 300-ms wait period.

Each subject completed a total of 1,080 trials consisting of six blocks of 180 trials. Stimuli for the whole-head condition and those for the eye-region condition were shown alternately in separate blocks. The order of the two conditions was counterbalanced across subjects. In each block, stimuli were presented in a random order, with four facial identities, five different head orientations (−30°, −15°, 0°, 15°, 30°), and nine different eye deviations (−20°, −15°, −10°, −5°, 0°, 5°, 10°, 15°, 20°). We use the term “eye deviation” to refer to the physical direction of the eyes relative to the observer. We reserve the term “gaze direction” for the subjective percept. Each observer repeated each condition three times.

#### Analysis

**Figure 3 fig3:**
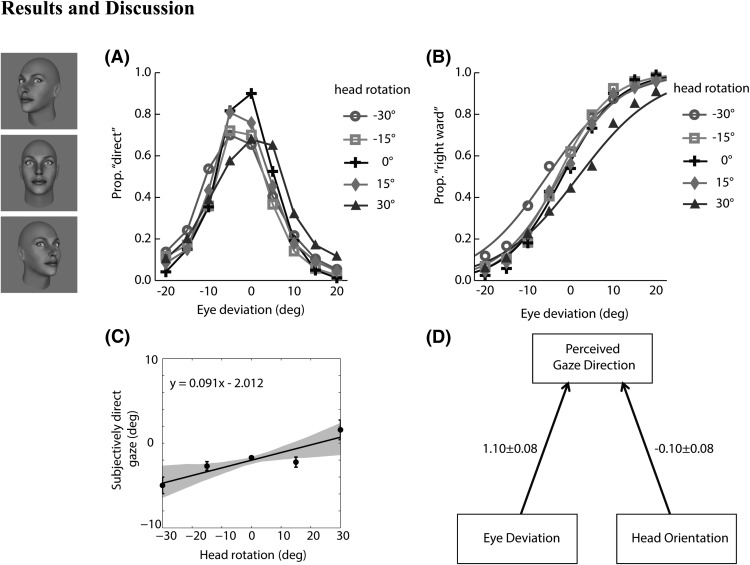
Data from the whole-head condition averaged across subjects. (A) Proportion of direct responses as a function of eye deviation for each head orientation. (B) Logistic fits to the data recoded as proportion of rightward response. (C) Points of subjectively direct gaze derived from the fitted data together with the linear regression slope across head orientation. The gray area represents bootstrapped 95% confidence intervals and the error bar represents the standard deviation between subjects. (D) Effective weights of eye deviation and head orientation on perceived gaze direction.

**Figure 4 fig4:**
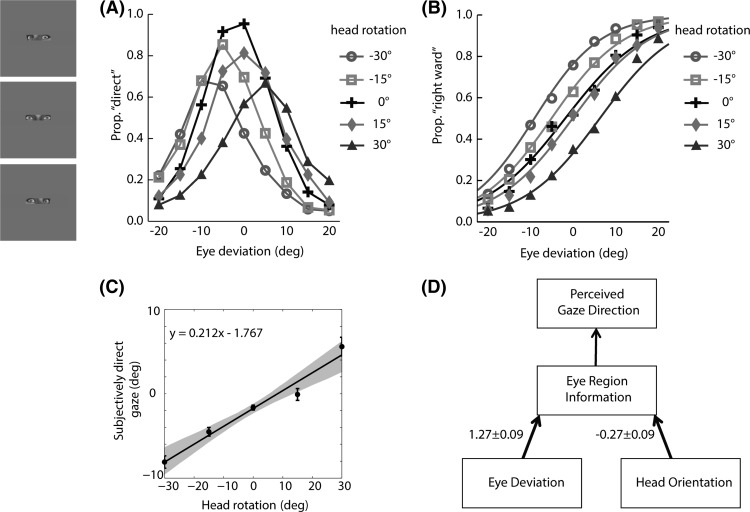
Data from the eye-region condition averaged across subjects. (A) Proportion of direct responses as a function of eye deviation for each head orientation. (B) Logistic fits to the data recoded as proportion of rightward response. (C) Points of subjectively direct gaze derived from the fitted data together with the linear regression slope across head orientation. The gray area represents bootstrapped 95% confidence intervals and the error bar represents the standard deviation between subjects. (D) Effective weights of eye deviation and head orientation on perceived gaze direction.

**Figure 5 fig5:**
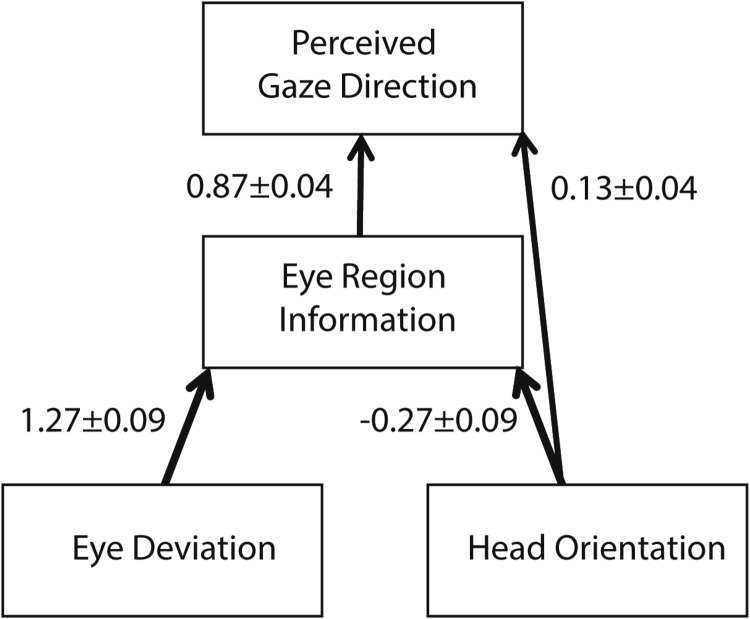
Dual-route model for the influence of head orientation on perceived gaze direction. The weights attached to each cue were derived by comparing the experimental results from the whole-head and eye-region conditions.

**Figure 6 fig6:**
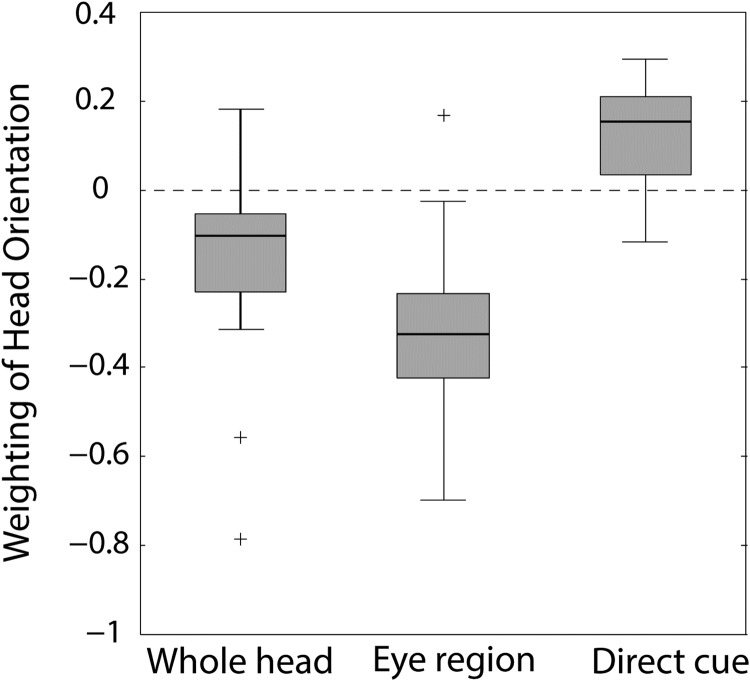
Box plot summarizing individual subjects’ (*n* = 20) overall weighting of head orientation in the whole-head and eye-region conditions, and the inferred weighting of head orientation as a direct cue in the whole-head condition. The box covers the interquartile range and the median is indicated by the mark within the box. The whiskers represent the most extreme data value within 1.5 times the interquartile range. Outlier values are depicted as +.

Subjects’ reports of direction of gaze as leftward, direct, or rightward were recoded as follows: leftward = 0; direct = 0.5; rightward = 1. A proportion rightward score for presentations of each head orientation and eye deviation was calculated as the sum of recoded scores divided by the number of presentations. The following analysis was performed both on the data averaged across subjects (results shown in Figures 3, 4, and 5) and on the individual data (results shown in Figure 6).[Fig-anchor fig3][Fig-anchor fig4][Fig-anchor fig5][Fig-anchor fig6]

For each head orientation, the proportion rightward score was fitted as a logistic function of eye deviation. The 50% point of each resulting psychometric function was taken as the eye deviation corresponding to subjectively direct gaze. On these points, we performed linear regression as a function of the degree of head rotation. The slope of the regression line, “m,” was used to estimate the relative weighting of eye deviation, “E,” and head orientation, “H” in determining perceived direction of gaze, “G.” The two weights were constrained to sum to one, such that

$$G\;=\;\left(\frac{1}{1\;-\;m}\right)E\;+\;\left(\frac{m}{m\;-\;1}\right)H.$$

Pairs of weights were derived separately for the whole-head and eye-region conditions. For the whole-head condition, the contributions of eye deviation and head orientation to perceived direction of gaze, G_WH_, were decomposed into a weighted combination of information from the eye region, G_ER_, and the effect of head orientation as a direct influence on perceived direction of gaze according to the following equation:

$$G_{WH}\;=\;\left(\frac{1\;-\;m_{ER}}{1\;-\;m_{WH}}\right)G_{ER}\;+\;\left(\frac{m_{WH}\;-\;m_{ER}}{m_{WH}\;-\;1}\right)H,$$

where m_WH_ and m_ER_ are the slopes of the regression lines from the whole-head and eye-region conditions, respectively.

In addition, individual data were analyzed by fitting the data to the psychophysical model developed by Mareschal et al. (2013b). For this analysis, the number of leftward, direct, and rightward response was counted for each gaze deviation at each head orientation.

### Results and Discussion

The results from the whole-head condition averaged across subjects are summarized in Figure 3. Figure 3A shows the proportion of “direct” responses as a function of eye deviation for each of the head orientation displays. Figure 3B shows the logistic fits to the data recoded as the proportion of the rightward responses for each of the head orientation displays. The eye deviation eliciting the 50% proportion rightward response from each psychometric function corresponds to the point of subjectively direct gaze for each head orientation. Figure 3C shows the points of subjectively direct gaze together with the linear regression slope across head orientation.

In general, the effect of head rotation is visible only with larger head rotation. For the larger amplitude of head rotation (±30°), there is an increase in “direct” responses for eye deviation in the same direction as the head rotation (i.e., the peaks of the direct responses shift in the direction of the head orientation). Similarly, the psychometric functions and the points of subjectively direct gaze tend to shift slightly toward the direction of head rotation. In addition, the points of subjectively direct gaze are not symmetrical around the physical 0° gaze point, but slightly shifted toward the left. Finally, from the slope of the regression line, we calculated the weights attached to the eye deviation and head orientation cues in direction of gaze perception (Figure 3D). The negative weight attached to head orientation indicates that the perceived direction of gaze is repelled from the orientation of the head in the whole-head condition.

The results from the eye-region condition averaged across subjects are summarized in Figure 4 in the same format as for the whole-head condition in Figure 3. The eye deviation eliciting the peak proportion of “direct” responses clearly shows a systematic shift toward the direction of head rotation (Figure 4A). The logistic fits to the proportion of rightward responses in the eye-region condition (Figure 4B) show a clear and systematic shift toward the direction of head rotation. The effect of the head rotation is to “repel” the perceived gaze direction from the head orientation. As in the whole-head condition, the points of subjectively direct gaze are shifted slightly toward the left (Figure 4C). Figure 4D illustrates how the eye deviation and head orientation cues are weighted in the perception of gaze direction when information is restricted to the eye region. As in the whole-head condition, the negative weight attached to head orientation indicates that the perceived direction of gaze is repelled from the orientation of the head.

When considering the results from two conditions together, the effect of head rotation found in both conditions was generally to “repel” the perceived gaze direction from head orientation. The magnitude of this effect was more pronounced for the eye-region condition. Based on the data from two conditions, we developed a dual-route model for the influence of head orientation on perceived gaze direction. Figure 5 illustrates how eye deviation and head orientation cues affect perceived gaze direction in the framework of the dual-route model together with the experimentally derived weights attached to each cue. Here, head orientation has a repulsive effect on the eye-region information, consistent with the change in the eccentricity of the iris within the visible part of the eye opening accompanying any change in the head orientation. This repulsive effect is illustrated by the negative weighting accompanying the arrow from head orientation to eye-region information in Figure 5. The repulsive effect of head orientation is reduced in the whole-head condition, suggesting that head orientation can also act as a direct cue to “attract” the perceived gaze direction toward head orientation. This attractive effect of head orientation is illustrated by the positive weighing accompanying the direct arrow from head orientation to perceived gaze direction in Figure 5.

All the analyses discussed this far concern data averaged across subjects. The analysis on individual data confirms the general trend. Figure 6 shows box plots depicting the weightings of head orientation for the whole-head and eye-region conditions, and inferred weighting of head orientation as a direct cue in the whole-head condition calculated for each observer. The *t* tests performed on the weightings of head orientation across subjects revealed significant differences from zero for all of these weights (whole head: *t*[19] = −3.02, *p* < .01, *d* = 0.68; eye region: *t*[19] = −7.42, *p* < .01, *d* = 1.66; direct cue: *t*[19] = 3.02, *p* < .01, *d* = 1.15). Again, head rotation affected perceived gaze direction in a consistent direction between the whole-head and eye-region conditions, but the effect was more pronounced for the eye-region condition. In both conditions, the perceived gaze direction was shifted in the opposite direction to the direction of head rotation (repulsive effect). The difference between the two conditions suggests that, when visible, head direction has an additional direct effect to “attract” the perceived gaze direction toward head orientation.

**Figure 7 fig7:**
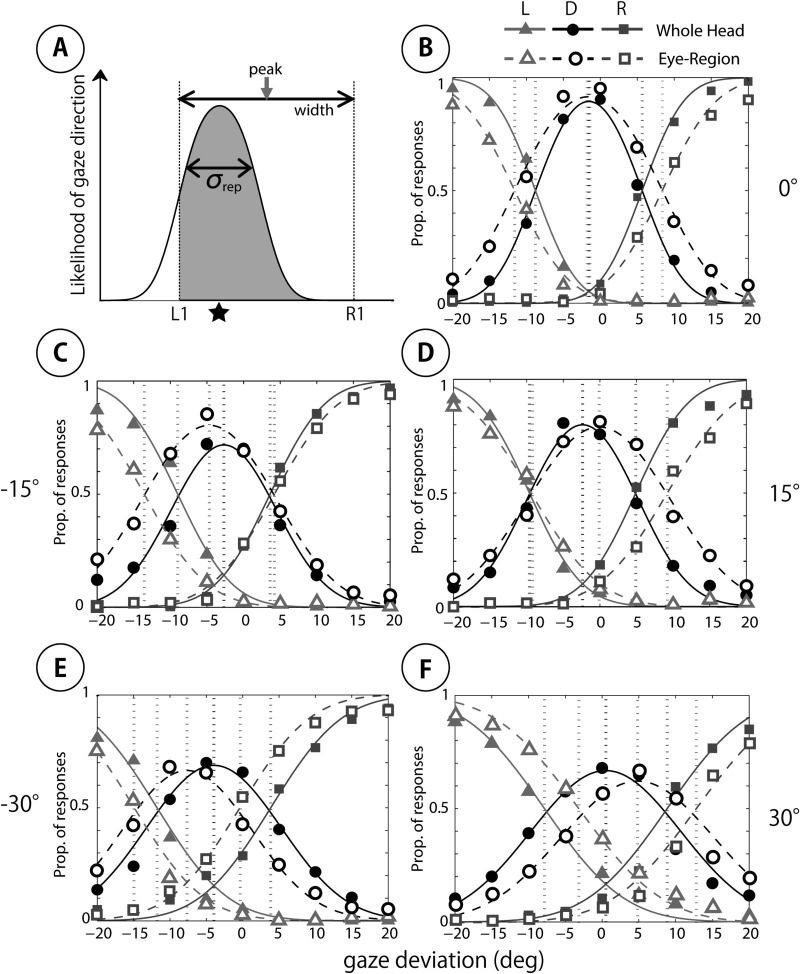
The psychophysical model of Mareschal et al. (2013b) and fit of the model to the categorization data averaged across subjects. (A) The psychophysical model showing an observer’s sensory representation of the gaze stimulus. The likelihood of the observer responding “direct” to the direction of gaze, indicated by the star, corresponds to the area of the gray region under the Gaussian. The likelihood of the observer responding “left” corresponds to the area of the white region, and the likelihood of responding “right” is effectively zero. The vertical dashed lines represent the categorical boundaries. The distance between the two represents the width of the cone of direct gaze. The middle point of the categorical boundaries is taken as the peak direction of perceptually direct gaze. The standard deviation of the likelihood function, σ_rep_, represents the level of sensory noise affecting the observer’s judgments (B to F). Model fit to the averaged data across subjects from the whole-head condition (solid lines) and from the eye-region condition (dashed lines) for each head orientation. The orientation of the head is represented by the number to the side of each panel. L = “left” response; D = “direct” response; R = “right” response.

To quantify the difference in gaze perception between the whole-head and eye-region conditions, we fitted the psychophysical model developed by Mareschal et al. (2013b) to the individual data. Figure 7A schematically represents the psychophysical model of Mareschal et al., and Figure 7B through 7F show the model fits to the data averaged across subjects for each head orientation in the whole-head and the eye-region conditions. Inspection of the bell-shaped curves representing “direct” response in each graph shows the peak response tends to shift toward the head orientation, and this trend is clearer for the eye-region (dashed line) condition than for the whole-head condition (solid line). In addition, the curve for the eye-region (dashed line) condition is wider than for the whole-head condition (solid line) at 0° head orientation, corresponding to more direct responses in the eye-region condition.[Fig-anchor fig7]

**Figure 8 fig8:**
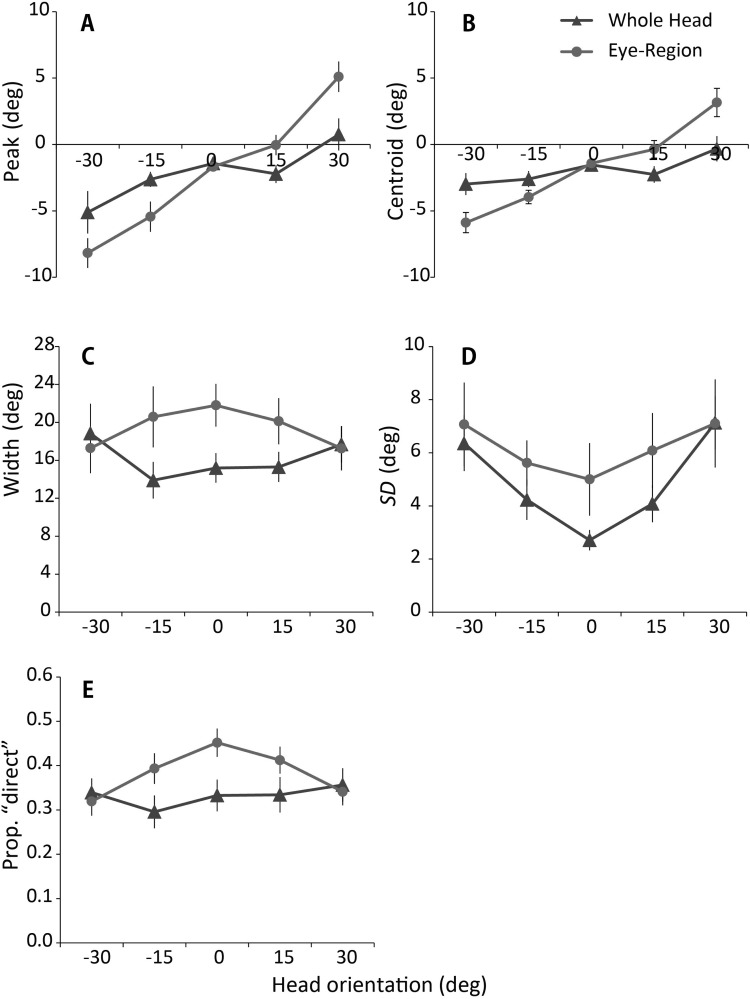
Measures of direct responding and fitted parameters from the model of Mareschal et al. (2013b). (A) Estimates of the midpoints (peaks) between the categorical boundaries obtained by fitting individual data to the psychophysical model of Mareschal et al. (B) The centroid of the direct responses. (C) The distances between the modeled categorical boundaries (widths). (D) The modeled standard deviations of the sensory noise. (E) The proportion of “direct” responses. Each value was computed individually, and averaged across subjects. Error bars represented ±1 standard error of the mean across subjects.

We fitted the model to individual subjects’ data and obtained for each an estimate of the peak direction of perceptually direct gaze (i.e., the midpoint between the fitted category boundaries for direct vs. averted gaze), an estimate of the width of the cone of direct gaze corresponding to the distance between the category boundaries (i.e., inverse specificity), and an estimate of the standard deviation of the noise affecting observers’ sensory representation of a gaze stimulus (i.e., inverse sensitivity). As an additional measure of subjectively direct gaze direction, we calculated the centroid of the “direct” gaze response as this would be less affected than the estimate of peak direction by the smaller number of trials performed in the current study compared with Mareschal et al. (2013b). Finally, we calculated the proportion of “direct” responses. The average of these estimates across subjects is shown in Figure 8. In the following analysis, we performed a repeated ANOVA with two conditions and five gaze deviations for each estimate.[Fig-anchor fig8]

The estimates of peak (Figure 8A) and centroid (Figure 8B) showed a pattern of results similar to those obtained from the analysis of the equilibrium of left and right gaze responses (Figure 3C vs. Figure 4C). Specifically, there was a greater repulsive effect of head orientation on perceived gaze direction for the eye-region than for the whole-head condition. For the estimate of peak (Figure 8A), there was a main effect of condition, *F*(4, 76) = 14.95, *p* < .01, η_p_^2^ = .44, and an interaction with head orientation, *F*(4, 76) = 15.00, *p* < .01, η_p_^2^ = .44. Simple effects analysis revealed significant effects of condition except at the 0° head rotation (−30°: *F*[1, 19] = 11.21, *p* < .01, η_p_^2^ = 0.37; −15°: *F*[1, 19] = 8.42, *p* < .01, η_p_^2^ = 0.31; 15°: *F*[1, 19] = 29.61, *p* < .01, η_p_^2^ = 0.34; 30°: *F*[1, 19] = 24.33, *p* < .01, η_p_^2^ = 0.56). Further, simple effects analysis showed an effect of head orientation for both the whole-head condition, *F*(4, 76) = 4.11, *p* < .01, η_p_^2^ = 0.18, and eye-region condition, *F*(4.76) = 27.39, *p* < .01, η_p_^2^ = 0.59.

As with the estimate of peak (Figure 8A), the analysis on the centroid (Figure 8B) showed a main effect of condition, *F*(4, 76) = 10.86, *p* < .01, η_p_^2^ = .36, interacting with head orientation, *F*(4, 76) = 15.24, *p* < .01, η_p_^2^ = .45. Simple effects analysis revealed significant effects of condition except at the 0° head rotation (−30°: *F*[1, 19] = 21.60, *p* < .01, η_p_^2^ = 0.53; −15°: *F*[1, 19] = 4.80, *p* < .01, η_p_^2^ = 0.20; 15°: *F*[1, 19] = 9.80, *p* < .01, η_p_^2^ = 0.34; 30°: *F*[1, 19] = 16.31, *p* < .01, η_p_^2^ = 0.46). However, the effect of head orientation was only shown for the eye-region condition, *F*(4.76) = 21.76, *p* < .01, η_p_^2^ = 0.53.

Unlike the estimates of peak (Figure 8A) and centroid (Figure 8B), other measures did not show a significant difference between the conditions at the extreme head angles of ±30°, suggesting that the shift in the peak direction of perceptually direct gaze due to the repulsive and attractive effects occurs independently to other aspects of performance change.

For the estimate of width (Figure 8C), there was a significant main effect of condition, *F*(1, 19) = 10.23, *p* < .01, η_p_^2^ = .35, interacting with head orientation, *F*(4, 76) = 13.39, *p* < .01, η_p_^2^ = .41. Simple effects analysis revealed a significant effect of condition, except at the extreme head angles of ±30° (−15°: *F*[1, 19] = 13.41, *p* < .01, η_p_^2^ = 0.41; 0°: *F*[1, 19] = 35.04, *p* < .01, η_p_^2^ = 0.65; 15°: *F*[1, 19] = 11.92, *p* < .01, η_p_^2^ = 0.39). In addition, the simple effects analysis showed the effect of head orientation both for the whole-head condition, *F*(4, 76) = 3.79, *p* < .01, η_p_^2^ = 0.17, and the eye-region condition, *F*(4.76) = 2.93, *p* < .05, η_p_^2^ = 0.13. Post hoc analysis for the whole-head condition showed that the width at −15° head orientation was significantly narrower than at 30° head orientation (*p* < .01). The post hoc analysis showed no significant difference for the eye-region condition.

For the estimate of standard deviation (Figure 8D), there was a significant main effect of head orientation, *F*(4, 76) = 18.15, *p* < .01, η_p_^2^ = .49. Simple effects analysis revealed a significant effect of condition, except at the extreme head angles of ±30° (−15°: *F*[1, 19] = 13.41, *p* < .01, η_p_^2^ = 0.41; 0°: *F*[1, 19] = 35.04, *p* < .01, η_p_^2^ = 0.65; 15°: *F*[1, 19] = 11.92, *p* < .01, η_p_^2^ = 0.39). Post hoc analysis with Shaffer’s sequential Bonferroni correction revealed a significant difference between 0° against all other head orientations (*p*s < .02), between −30° and −15° (*p* < .02), and between 15° and 30° (*p* < .01). These results suggest a generally higher sensitivity to gaze direction around the frontal head orientation irrespective of the image conditions.

For the proportion of “direct” gaze responses (Figure 8E), there was a main effect of condition, *F*(1, 19) = 15.81, *p* < .01, η_p_^2^ = .45, a main effect of head orientation, *F*(4, 76) = 6.29, *p* < .01, η_p_^2^ = .24, and a significant interaction, *F*(4, 76) = 15.19, *p* < .01, η_p_^2^ = .44. Simple effects analysis revealed significant effects of condition, except at the extreme head angles of ±30° (−15°: *F*[1, 19] = 21.01, *p* < .01, η_p_^2^ = 0.53; 0°: *F*[1, 19] = 39.20, *p* < .01, η_p_^2^ = 0.67; 15°: *F*[1, 19] = 39.20, *p* < .01, η_p_^2^ = 0.40). In addition, the simple effects analysis showed an effect of head orientation both for the whole-head condition, *F*(4, 76) = 3.12, *p* < .02, η_p_^2^ = 0.14, and the eye-region condition, *F*(4, 76) = 5.06, *p* < .05, η_p_^2^ = 0.44. The difference in the whole-head condition was due to somewhat fewer “direct” responses at −15°; post hoc analysis showed significant differences from −30° (*p* < .05), 15°(*p* < .02), and 30°(*p* < .01). In the eye-region condition, the proportion of “direct” responses was greater around the frontal head orientation compared with more angled orientations. The post hoc analysis showed significant differences in the proportion of “direct” responses between 0° and −15°(*p* < .05), between 0°and ±30°(*p* < .01), between 15° and ±30°(*p* < .01), and between −15° and −30° (*p* < .01). No other effect or interaction reached significance.

## Control Experiment

In the main experiment, we found that the repulsive effect was reduced in the whole-head condition compared with the eye-region condition across various measures of subjectively direct gaze. In addition, we found that the proportion of “direct gaze” responses increased for the eye-region compared with the whole-head condition, which seems to run counter to the reduction in “direct” responses for eye-only stimuli reported by Mareschal et al. (2013b). The variation in procedure and stimuli between these studies may account for the differential results. First, faces in various head orientations were interleaved in the current study, whereas facial orientation was fixed to straight ahead in Mareschal et al. Second, facial information around the eyes was included in the current eye-region stimuli but not in those used by Mareschal et al.

Previous studies have reported that high uncertainty tends to lead observers to report gaze as being direct (Mareschal et al. 2013a; Martin & Jones, 1982; Martin & Rovira, 1981). Based on these findings, we speculate that high uncertainty about head orientation in the eye-region display of the current study have induced the greater number of direct responses in this condition. Unlike Mareschal et al. (2013a, 2013b), who manipulated the uncertainty (visibility) of eyes by adding noise, the current study employed no such manipulation and the eyes themselves were clearly visible across conditions. Therefore, uncertainty imposed on the eyes is unlikely to explain the current results. Instead, the pattern of results could be related to uncertainty about head orientation. As the images in various head orientations were shown in random order in the current study, the participants had to estimate the orientation of head as well as the direction of gaze based on the stimulus image on each trial. The occlusion of head context in the eye-region condition would have made the uncertainty about head orientation higher for this condition than in the whole-head condition. However, we found that the increase in the number of direct responses was limited to the frontal head orientation. We speculate that perspective cues, together with a clearly oriented nose bridge, provided clear enough information about head orientation to overcome any such uncertainty for extreme angles.

**Figure 9 fig9:**
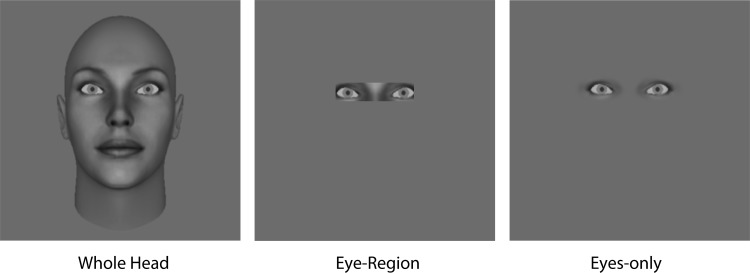
Example of stimulus images used in the control experiment. All images were in the frontal head orientation.

This interpretation suggests that the number of “direct gaze” responses would be similar between the whole-head and eye-region conditions if the uncertainty about head orientation was removed. Here, we explicitly tested this possibility by conducting a control experiment in which only the frontal (0°) head orientation was shown, thereby eliminating the uncertainty about head orientation. We chose the frontal head orientation because the difference in the number of direct responses was most pronounced in this condition in the main experiment. In addition, we included an eye-only version of the stimuli, as employed in Mareschal et al. (2013b), to examine the effect of including the nose bridge (see Figure 9).[Fig-anchor fig9]

### Method

#### Participants

Twenty naïve observers (8 male and 12 female) served as subjects (mean age = 19.75 years; *SD* = 2.24 years). All had normal or corrected normal vision. All experiments adhered to the Declaration of Helsinki (2008) guidelines and were approved by the University of Sydney Human Research Ethics Committee.

#### Apparatus, stimuli, and procedure

Apparatus, stimuli, and procedure were the same as in the main experiment, except for the following: We created eyes-only images, as in Mareschal et al. (2013b), by applying an elliptical raised cosine contrast envelope over each eye. Each subject performed 108 trials consisting of three blocks of 36 trials for each of three conditions: whole-head condition, eye-region condition, and eyes-only condition. Unlike in the main experiment, the orientation of the head was fixed to 0°. The images in the three conditions were shown in separate blocks, with images in one condition being shown in three consecutive blocks. The order of the conditions was randomized between subjects.

### Results and Discussion

**Figure 10 fig10:**
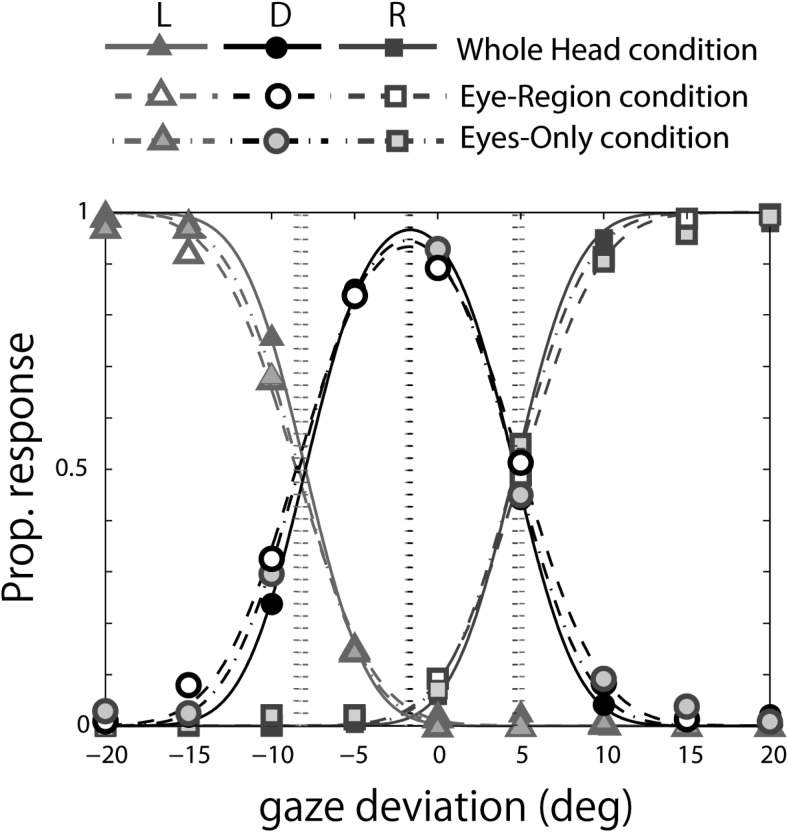
Fit of the model of Mareschal et al. (2013b) to control experiment data. The categorization data at 0° head orientation averaged across subjects fitted by the model. L = “left” response; D = “direct” response; R = “right” response.

As with the main experiment, we fitted the psychophysical model developed by Mareschal et al. (2013b) to the individual data. Figure 10 shows the data averaged across subjects fitted by the Mareschal et al. method. Unlike the data from the main experiment (Figure 7B), there is little difference between the conditions in the control experiment.[Fig-anchor fig10]

**Figure 11 fig11:**
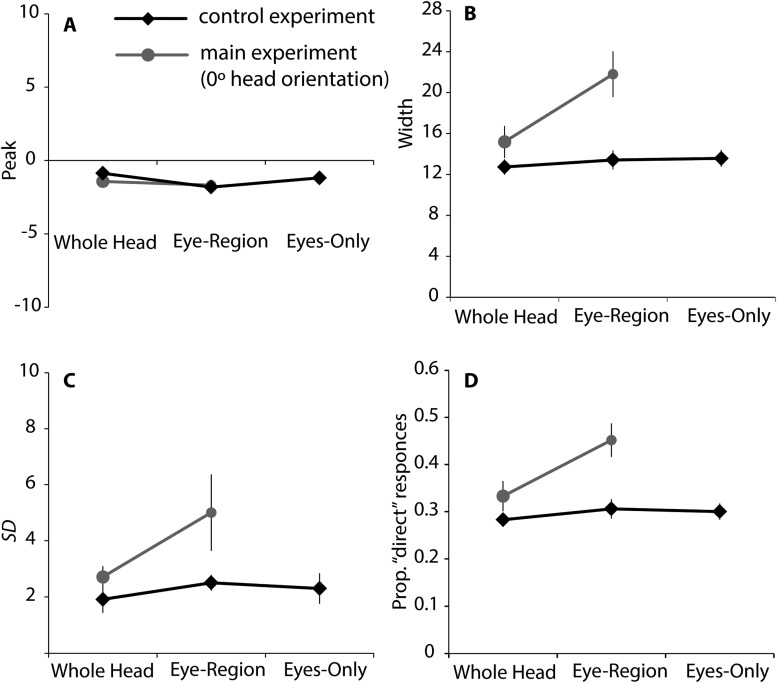
Results from the control experiment together with the results from the main experiment at 0° head orientation. Estimates of peaks (A), widths (B), and standard deviations (C) in the whole-head and eye-region conditions based on the model by Mareschal et al. (2013b), and the proportion of “direct” responses (D). Averaged data across subjects are shown. Error bars represented ±1 standard error of the mean across subjects.

Figure 11 shows the estimate of the peak direction of perceptually direct gaze (A; i.e., the midpoint between the fitted category boundaries for direct vs. averted gaze), the estimate of the width of direct judgments (B) corresponding to the distance between the category boundaries (i.e., inverse specificity), and the estimate of the standard deviation of the observers’ sensory representation of a gaze stimulus (C; i.e., inverse sensitivity), together with the proportion of “direct” responses (D). A one-way repeated ANOVA revealed no significant difference between the conditions for any of these estimates.[Fig-anchor fig11]

To compare the data from the main experiment at 0° head orientation with those from the control experiment, we performed a two-way ANOVA with condition (whole-head, eye-region) as the repeated factor and experiment (main experiment, control experiment) as the between subject factor.

The ANOVA on the proportion of “direct” responses (Figure 11D) revealed a significant main effect of condition, *F*(1, 38) = 7.12, *p* < .02, η_p_^2^ = 0.15, experiment, *F*(1, 38) = 39.00, *p* < .01, η_p_^2^ = 0.51, and a significant interaction, *F*(1, 38) = 17.73, *p* < .01, η_p_^2^ = 0.32. Simple effects analysis showed that the proportion of “direct” responses was significantly greater in the main experiment than in the control experiment only in the eye-region condition, *F*(1, 38) = 12.60, *p* < .01, η_p_^2^ = 0.24. Similarly, the proportion of “direct” responses was significantly greater in the eye-region condition than in the whole-head condition only in the main experiment, *F*(1, 19) = 39.20, *p* < .01, η_p_^2^ = 0.24.

Similarly, the ANOVA on the estimate of the width of the cone of direct gaze (Figure 11B) revealed significant main effects of condition, *F*(1, 38) = 7.24, *p* < .02, η_p_^2^ = 0.16, experiment, *F*(1, 38) = 32.76, *p* < .01, η_p_^2^ = 0.46, and their interaction, *F*(1, 38) = 21.53, *p* < .01, η_p_^2^ = 0.36. Simple effects analysis showed that the width was significantly greater in the main experiment than in the control experiment only in the eye-region condition, *F*(1, 38) = 11.89, *p* < .01, η_p_^2^ = 0.24, and that the width was significantly greater in the eye-region condition than in the whole-head condition only in the main experiment, *F*(1, 19) = 35.04, *p* < .01, η_p_^2^ = 0.65.

Finally, for the estimate of the standard deviation, the ANOVA revealed a significant main effect of experiment (Figure 11C), *F*(1, 38) = 11.89, *p* < .01, η_p_^2^ = 0.24, showing a smaller standard deviation in the control experiment than in the main experiment, and a main effect of condition, *F*(1, 38) = 4.91, *p* < .05, η_p_^2^ = 0.11, showing a greater standard deviation in the eye-region than in the whole-head condition. No other effect or interaction reached significance.

The similarity in results between conditions in the control experiment is consistent with the interpretation that, in the main experiment, the greater number of “direct” responses in the eye-region than in the whole-head condition was due to higher uncertainty about head orientation in the eye-region condition. The smaller estimated standard deviation in the control experiment than in the main experiment argues against the possibility that generally worse performance in this condition could account for the lack of any difference in the proportion of “direct” responses between the conditions in the current experiment.

Although we introduced an eyes-only condition, we did not replicate the decrease in the proportion of “direct” responses in this condition compared with the whole-head condition reported in Mareschal et al. (2013b). Remaining differences between the studies include a wider range of gaze deviations and a smaller number of trials in the current study compared with Mareschal et al. However, we are unsure how these can explain the lack of any tendency for the proportion of “direct” responses to decrease in the eyes-only condition in the current study. Although Mareschal et al. tested the two conditions as independent experiments, we tested all three conditions on the same occasion. In the current study, the trials from different conditions were thus performed at close temporal proximity. This might have encouraged our subjects to apply the same criteria to judge “direct” gaze across the conditions.

## General Discussion

**Figure 12 fig12:**
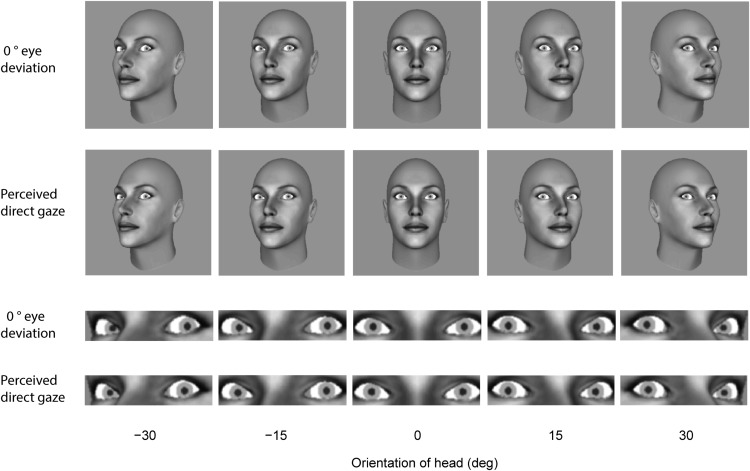
Illustration of 0° eye deviation (physically direct gaze) and the eye deviation corresponding to perceived direct gaze according to the weightings computed from the mean data across subjects for each head orientation in the whole-head and the eye-region display conditions.

By comparing perceived gaze direction in the whole-head condition and in the eye-region condition, the current study revealed two routes whereby head orientation affects perceived gaze direction. In general, we found that lateral head rotation tends to have a repulsive effect on gaze perception, in which the perceived gaze direction is biased in the opposite direction to head orientation (e.g., the eyes might need to be deviated by +5° in a +30° rotated head to overcome the repulsive effect of the head and be seen as direct). The repulsive effect is consistent with the effect of head rotation on perceived gaze direction observed in previous studies that used real human faces or realistic 3D head models as stimuli (Anstis et al., 1969; Gamer & Hecht, 2007; Gibson & Pick, 1963; Noll, 1976). As pointed out by Anstis et al., turning the head with gaze fixed on a given point (e.g., directly ahead) changes the visible part of the eye on either side of the iris. As the head rotates to the right, for example, the relative amount of visible white (sclera) on the right side of the iris increases, just like when eye direction shifts toward the left (see Figure 12).[Fig-anchor fig12]

The magnitude of the repulsive effect was more pronounced in the eye region condition, in which little information about the orientation of the head is available, than in the whole-head condition, in which reliable information about head orientation is available (this is most visualized by comparing the eye regions on the ±30° head rotated stimuli in Figure 12). Thus, change in the information available from the eye region appears to be the primary cause of the repulsive effect of head rotation. The reduction of the repulsive effect in the whole-head condition indicates that head orientation itself has a direct influence on perceived gaze direction in a manner that attracts perceived gaze toward the head orientation. This attractive effect of head orientation on perceived gaze direction is consistent with that observed in the Wollaston effect (Wollaston, 1824) and previous studies using stimuli consisting of identical eyes placed in different facial contexts (Langton et al., 2004; Maruyama & Endo, 1983; Todorović, 2006, 2009). Considering that the primary cause of the repulsive effect is the change in the information within the eye region, the placement of identical eyes in various head orientation contexts would eliminate the repulsive effect of head rotation and maximize the attraction effect. In the case of a more realistic situation in which the visible eye region changes with head orientation, the attraction effect would act to compensate for the biased information obtained from the angled eye region.

Unlike the studies mentioned above, Cline (1967) reported an attractive effect of head orientation when using real faces as stimuli. In his study, perceived gaze direction was constantly biased toward the head orientation when the head was rotated rightward by 30°. In some of his experiments, a constant bias in perceived gaze direction toward the right was also reported in the case of a frontal face. Examination of the figure describing his experimental setting shows that the face was illuminated from the left. Asymmetrical lighting is known to produce a shift in apparent facial orientation opposite to the light source (Troje & Siebeck, 1998). Further, the lighting from the left side of the face might have reduced the relative visibility of the sclera to the right of the iris that would likely induce an apparent shift of gaze direction toward the right. As Cline did not counter balance the direction of head orientation, his results may have been confounded with the effect of asymmetrical lighting.

By fitting the psychophysical model developed by Mareschal et al. (2013b) to our data, we have further quantified gaze perception in the whole-head and eye-region conditions. The results from this analysis showed that the repulsive effect of head orientation occurs independently to other aspects of performance change. The estimate of peak direction of perceptually direct gaze (Figure 8A) showed the same pattern of results as we obtained with the analysis of equilibrium of left and right gaze response (Figure 3C vs. Figure 4C). Although these measures showed that the difference in the magnitude of repulsive effect between the whole-head and eye-region conditions was greatest in the extreme angle of ±30°, no other measure showed any difference between the conditions at these head orientations. On the contrary, some of the measures showed significant differences around the frontal head orientation. In particular, there was an increase in the number of direct responses in the eye-region condition relative to the whole-head condition. We interpreted the increase in the number of direct responses in the eye-region condition as a consequence of uncertainty about head orientation in this condition, especially around the frontal head orientation (0° and ±15°). In fact, the results from the control experiment confirmed that no such increase in the number of direct responses occurred when the uncertainty about head orientation was eliminated.

In seminal work on the neural basis of gaze perception, Perrett et al. (Perrett, Hietanen, Oram, & Benson, 1992; Perrett et al., 1985) reported that most of the cells in macaque superior temporal sulcus (STS) that are sensitive to head orientation are also sensitive to gaze direction. Such cells sensitive to both head and gaze direction are likely to mediate the process of integrating information from eye region and head orientation. Consistent with this, a recent fMRI study revealed that anterior STS in humans codes others’ gaze direction in a manner invariant across head orientation (Carlin, Calder, Kriegeskorte, Nili, & Rowe, 2011). Perrett et al. (1992) proposed that sensitivity to eye gaze overrides sensitivity to head view, based on the finding that preferential responses to particular head orientations by cells in macaque STS were modulated by the direction of eye gaze. However, they also suggested that head orientation provides a useful default cue to the direction of others’ attention when eyes are not clearly visible (e.g., when observed from a distance or when strong light from above casts a shadow around the eyes), as they found that the cells showed sensitivity to head orientation even when the eyes were occluded.

In this article, we have revealed a more subtle way in which information from the eye region is integrated with head orientation to arrive at the perceived gaze direction. Consistent with the discussion by Perrett et al. (1992), we assume that the weights attached to each cue would not be fixed but would vary depending on viewing conditions and the information available in the display (i.e., increased uncertainty for one cue is likely to reduce the relative weight attached to that cue). In fact, Gamer and Hecht (2007) reported that the repulsive effect was greater at the viewing distance of 1 m than at 5 m. Considering that a greater weighting of eye-region information in the current framework would result in a greater repulsive effect, the results of Gamer and Hecht are consistent with the idea that the better visibility of the irises and pupils at closer viewing distance results in greater weighting of eye-region information. Although the eyes were always clearly visible in the current study, uncertainty in the deviation of the eyes associated with low visibility leads observers to tend to report gaze as being direct (Mareschal et al., 2013a). Thus, reduction in the visibility of the eyes may reveal the influence of a prior bias for direct gaze in addition to reducing the weighting of eye-region information.

The relative weighting of eye-region information and head orientation could also vary between the individuals. As discussed by Mareschal et al. (2013b), the gaze categorization methodology employed in the current study can be applied to developmental (Vida & Maurer, 2012) and clinical populations, such as people with autism who show atypical gaze processing (Campbell et al., 2006; Pellicano, Rhodes, & Calder, 2013; Senju, Yaguchi, Tojo, & Hasegawa, 2003; see also Webster & Potter, 2008). Both young children and autistic individuals tend to show superiority in the processing of local over global visual information, unlike adults and typical individuals (e.g., Scherf, Luna, Kimchi, Minshew, & Behrmann, 2008). Accordingly, they might place greater reliance on eye-region information in judging perceived gaze direction. If so, they should be more susceptible to the repulsive effect of head rotation and particularly prone to inaccurate judgment of gaze direction for faces viewed from an angle. Further studies will reveal how the relative weightings of eye-region information and head orientation vary with changes to the information available in the retinal image of the observer, and how they vary between clinical populations and controls.

Finally, we note that the precise value of the weight for the direct cue reported in the current study (0.13) might tend to be an underestimate. This is because the weight was derived from the difference in performance between the whole-head and the eye-region conditions. If the direct cue of head orientation were not entirely abolished in the eye-region condition, then this would cause the weights attached to the “direct route” in the model to be underestimated. It is possible that the inclusion of the bridge of the nose in the eye-region condition served as a cue to head orientation, reducing the difference from the whole-head condition and hence causing the weight attached to the direct cue of head orientation to be underestimated.

In conclusion, we found that although head orientation generally induced a repulsive effect, its magnitude was reduced in the whole-head compared with the eye-region condition. This reduction suggests that, in the whole-head condition, an attractive effect of head orientation acts to compensate for the repulsive effect induced from the angled eye region, reducing the error in the resultant perceived gaze direction. The dual-route model developed based on these results provides the first integrative framework to understand the relationship between these two effects of head orientation on perceived gaze direction.

## Figures and Tables

**Figure 1 fig1:**
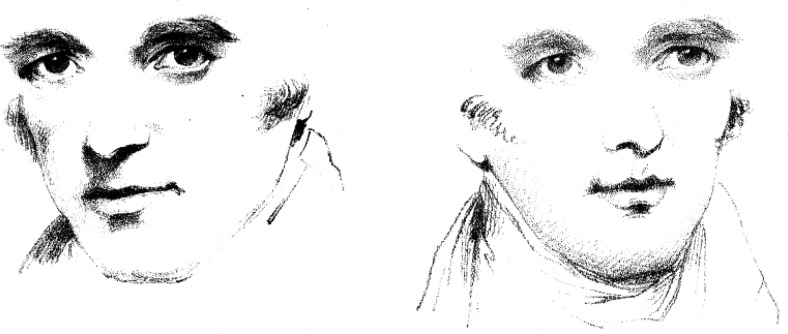
Demonstration by Wollaston (From “On the Apparent Direction of Eyes in a Portrait,” by W. H. Wollaston, 1824, *Philosophical Transactions of the Royal Society of London, 114*, p. 256. In the public domain). From the drawing of a face oriented leftward with direct gaze (left), Wollaston produced another face by inserting the same eyes into a drawing of the same individual with his head oriented to the right (right). Although these two faces share identical eyes, the latter appears to be looking to the right of the viewer.

## References

[c1] Anstis S. M., Mayhew J. W., & Morley T. (1969). Perception of where a face or television “portrait” is looking. The American Journal of Psychology, 82, 474–489. doi:10.2307/1420441 5398220

[c2] Brainard D. H. (1997). The psychophysics toolbox. Spatial Vision, 10, 433–436. doi:10.1163/156856897X00357 9176952

[c3] Campbell R., Lawrence K., Mandy W., Mitra C., Jeyakuma L., & Skuse D. (2006). Meanings in motion and faces: Developmental associations between the processing of intention from geometrical animations and gaze detection accuracy. Development and Psychopathology, 18, 99–118. doi:10.1017/S0954579406060068 16478554

[c4] Carlin J. D., Calder A. J., Kriegeskorte N., Nili H., & Rowe J. B. (2011). A head view-invariant representation of gaze direction in anterior superior temporal sulcus. Current Biology, 21, 1817–1821. doi:10.1016/j.cub.2011.09.025 22036180 PMC3267037

[c5] Cline M. G. (1967). The perception of where a person is looking. The American Journal of Psychology, 80, 41–50. doi:10.2307/1420539 6036357

[c6] Ewbank M. P., Jennings C., & Calder A. J. (2009). Why are you angry with me? Facial expressions of threat influence perception of gaze direction. Journal of Vision, 9, 16. doi:10.1167/9.12.16 20053107

[c7] Gamer M., & Hecht H. (2007). Are you looking at me? Measuring the cone of gaze. Journal of Experimental Psychology: Human Perception and Performance, 33, 705–715. doi:10.1037/0096-1523.33.3.705 17563231

[c8] Gibson J. J., & Pick A. D. (1963). Perception of another person’s looking behavior. The American Journal of Psychology, 76, 386–394. doi:10.2307/1419779 13947729

[c9] Kluttz N. L., Mayes B. R., West R. W., & Kerby D. S. (2009). The effect of head turn on the perception of gaze. Vision Research, 49, 1979–1993. doi:10.1016/j.visres.2009.05.013 19467254

[c10] Kobayashi H., & Kohshima S. (1997). Unique morphology of the human eye. Nature, 387, 767–768. doi:10.1038/42842 9194557

[c11] Kobayashi H., & Kohshima S. (2001). Unique morphology of the human eye and its adaptive meaning: Comparative studies on external morphology of the primate eye. Journal of Human Evolution, 40, 419–435. doi:10.1006/jhev.2001.0468 11322803

[c12] Langton S. R. (2000). The mutual influence of gaze and head orientation in the analysis of social attention direction. The Quarterly Journal of Experimental Psychology Section. A, Human Experimental Psychology, 53, 825–845. doi:10.1080/713755908 10994231

[c13] Langton S. R., Honeyman H., & Tessler E. (2004). The influence of head contour and nose angle on the perception of eye-gaze direction. Perception & Psychophysics, 66, 752–771. doi:10.3758/BF03194970 15495901

[c14] Lobmaier J. S., Tiddeman B. P., & Perrett D. I. (2008). Emotional expression modulates perceived gaze direction. Emotion, 8, 573–577. doi:10.1037/1528-3542.8.4.573 18729587

[c15] Mareschal I., Calder A. J., & Clifford C. W. G. (2013a). Humans have an expectation that gaze is directed toward them. Current Biology, 23, 717–721. doi:10.1016/j.cub.2013.03.030 23562265 PMC3918857

[c16] Mareschal I., Calder A. J., Dadds M. R., & Clifford C. W. (2013b). Gaze categorization under uncertainty: Psychophysics and modeling. Journal of Vision, 13, 18. doi:10.1167/13.5.18 23608340

[c17] Martin W. W., & Jones R. F. (1982). The accuracy of eye-gaze judgment: A signal detection approach. British Journal of Social Psychology, 21, 293–299. doi:10.1111/j.2044-8309.1982.tb00551.x 7171930

[c19] Martin W., & Rovira M. (1982). Response biases in eye-gaze perception. The Journal of Psychology: Interdisciplinary and Applied, 110, 203–209. doi:10.1080/00223980.1982.9915341 7069638

[c18] Martin W. W., & Rovira L. M. (1981). An experimental analysis of discriminability and bias in eye-gaze judgment. Journal of Nonverbal Behavior, 5, 155–163. doi:10.1007/BF00986132

[c20] Maruyama K., & Endo M. (1983). The effect of face orientation upon apparent direction of gaze. Tohoku Psychologica Folia, 42, 126–138

[c21] Noll A. M. (1976). The effects of visible eye and head turn on the perception of being looked at. The American Journal of Psychology, 89, 631–644. doi:10.2307/1421462 1020765

[c22] Pellicano E., Rhodes G., & Calder A. J. (2013). Reduced gaze aftereffects are related to difficulties categorising gaze direction in children with autism. Neuropsychologia, 51, 1504–1509. doi:10.1016/j.neuropsychologia.2013.03.021 23583965 PMC3708125

[c23] Perrett D. I., Hietanen J. K., Oram M. W., & Benson P. J. (1992). Organization and functions of cells responsive to faces in the temporal cortex. Philosophical Transactions of the Royal Society of London Series B-Biological Sciences, 335, 23–30. doi:10.1098/rstb.1992.0003 1348133

[c24] Perrett D. I., Smith P. A. J., Potter D. D., Mistlin A. J., Head A. S., Milner A. D., & Jeeves M. A. (1985). Visual cells in the temporal cortex sensitive to face view and gaze direction. Proceedings of the Royal Society of London. Series B, Containing Papers of a Biological Character. Royal Society (Great Britain), 223, 293–317. doi:10.1098/rspb.1985.0003 2858100

[c25] Ricciardelli P., & Driver J. (2008). Effects of head orientation on gaze perception: How positive congruency effects can be reversed. Quarterly Journal of Experimental Psychology (2006), 61, 491–504. doi:10.1080/17470210701255457 17853198

[c26] Scherf K. S., Luna B., Kimchi R., Minshew N., & Behrmann M. (2008). Missing the big picture: Impaired development of global shape processing in autism. Autism Research, 1, 114–129. doi:10.1002/aur.17 19360658 PMC2670479

[c27] Senju A., Yaguchi K., Tojo Y., & Hasegawa T. (2003). Eye contact does not facilitate detection in children with autism. Cognition, 89, B43–B51. doi:10.1016/S0010-0277(03)00081-7 12893128

[c28] Seyama J., & Nagayama R. (2005). The effect of torso direction on the judgement of eye direction. Visual Cognition, 12, 103–116. doi:10.1080/13506280444000111

[c29] Slepian M. L., Weisbuch M., Adams R. B., & Ambady N. (2011). Gender moderates the relationship between emotion and perceived gaze. Emotion, 11, 1439–1444. doi:10.1037/a0026163 22142212

[c30] Stoyanova R. S., Ewbank M. P., & Calder A. J. (2010). “You talkin’ to me?”: Self-relevant auditory signals influence perception of gaze direction. Psychological Science, 21, 1765–1769. doi:10.1177/0956797610388812 21078896

[c31] Todorović D. (2006). Geometrical basis of perception of gaze direction. Vision Research, 46, 3549–3562. doi:10.1016/j.visres.2006.04.011 16904157

[c32] Todorović D. (2009). The effect of face eccentricity on the perception of gaze direction. Perception, 38, 109–132. doi:10.1068/pp.5930 19323141

[c33] Troje N. F., & Siebeck U. (1998). Illumination-induced apparent shift in orientation of human heads. Perception, 27, 671–680. doi:10.1068/pp.270671 10197186

[c34] Vida M. D., & Maurer D. (2012). Gradual improvement in fine-grained sensitivity to triadic gaze after 6 years of age. Journal of Experimental Child Psychology, 111, 299–318. doi:10.1016/j.jecp.2011.08.009 21982081

[c35] Webster S., & Potter D. D. (2008). Brief report: Eye direction detection improves with development in autism. Journal of Autism and Developmental Disorders, 38, 1184–1186. doi:10.1007/s10803-008-0539-9 18324465

[c36] Wollaston W. H. (1824). On the apparent direction of eyes in a portrait. Philosophical Transactions of the Royal Society of London, 114, 247–256. doi:10.1098/rstl.1824.0016

[c37] World Medical Association (2008). Declaration of Helsinki–Ethical Principles for Medical Research involving human subjects. As amended by the 59th WMA General Assmbly, Seoul, October 19886379

